# Botulinum toxin combined with static progressive stretching improves fibrous stiffness of knee joint in rats through TGF-β1/Smad pathway

**DOI:** 10.17305/bb.2024.10526

**Published:** 2024-07-22

**Authors:** Xin He, Xin Zhang, Xin Zhao, Xiaoju Li, Ke Chen, Yingying Liao, Xiechen Feng, Yiyan Zou

**Affiliations:** 1 The Third People’s Hospital of Chengdu, Chengdu, China; 2 Sichuan Provincial Orthopedic Hospital, Chengdu, China; 3 Sichuan Electric Power Hospital, Chengdu, China; 4 School of Sports Medicine and Health, Chengdu Sport University, Chengdu, China; 5 Sichuan Provincial Rehabilitation Hospital, Chengdu, China

**Keywords:** Knee joint stiffness, joint capsule fibrosis, botulinum toxin type A (BTX-A), static progressive stretching (SPS), TGF-β1/small mother against decapentaplegic (Smad) pathway

## Abstract

Joint stiffness and fibrosis are common complications that affect mobility and quality of life, necessitating effective therapeutic strategies to alleviate these issues. The study aimed to observe the therapeutic effect of static progressive stretching (SPS) combined with botulinum toxin type A (BTX-A) on knee joint stiffness in rats and its effect on the transforming growth factor beta 1 (TGF-β1)/small mother against decapentaplegic (Smad) pathway in the development of joint capsule fibrosis. Forty Sprague–Dawley (SD) rats were randomly divided into the blank control group, model control group, SPS intervention group, BTX-A intervention group, and SPS combined with BTX-A intervention group. Except for the blank control group, the right knee joints of the other rats were surgically fixed with Kirschner wire internal immobilization in full flexion for four weeks to form joint flexion contracture and cause fibrotic stiffness of the joint. The therapeutic effect of each intervention was assessed by the range of motion (ROM) of the knee joint, joint stiffness, the number of total cells, and collagen deposition in the posterior joint capsule, as well as the protein level expressions of TGF-β1, Smad2, Smad3, Smad4, p-Smad2/3, collagen I and III, and alpha-smooth muscle actin (α-SMA) in the posterior joint capsule in the TGF-β1/Smad pathway. SPS combined with BTX-A was more effective in relieving joint fibrosis stiffness, improving the histopathological changes in the posterior joint capsule, and suppressing the high expression of target proteins and the overactivated TGF-β1/Smad pathway. The overactivated TGF-β1/Smad pathway was involved in the formation of knee joint fibrosis stiffness in rats. SPS combined with BTX-A was effective in relieving joint flexion contracture and fibrosis of the joint capsule. Moreover, the inhibition of the overactivated TGF-β1/Smad pathway may be the potential molecular mechanism for its therapeutic effect.

## Introduction

Joint stiffness often occurs as a result of muscular and soft tissue contraction, adhesion, and fibrosis surrounding the joint after traumas inside or outside the joint. The affected limbs display flexion contracture and/or extension contracture in comparison to the healthy side, leading to a permanent restriction in the range of motion (ROM) [1–3]. Knee joint stiffness is a common type of joint stiffness. Previous studies have shown that knee joint stiffness commonly occurs following trauma and postoperative operations. The prevalence of knee joint stiffness is 5.3% after total knee replacement, 0%–4% after knee ligament restoration, and 21.1% after knee joint fracture [4, 5]. Joint stiffness is mostly caused by the presence of fibrosis in the joint capsule. This syndrome is characterized by inflammation, the accumulation of extracellular matrix (ECM), increased collagen synthesis, and the disturbance of collagen alignment [6]. Botulinum neurotoxin (BTX) is a protein neurotoxin produced by the anaerobic strain of Clostridium bacteria [7]. The single-chain polypeptide in question is inactive and has a molecular weight of approximately 150 kD. Within BTX, there are seven distinct serotypes: A, B, C, D, E, F, and G. The inhibition of acetylcholine release from nerve terminals is observed across all serotypes, but there are significant variations in their intracellular target proteins, properties of action, and resulting effects. Currently, Botulinum toxin type A (BTX-A) is the most extensively researched serotype. The effectiveness of this substance is highly significant and specific to the neurologic system. However, its diffusion is restricted when administered locally, and its impact gradually diminishes over time [8]. Studies have shown that BTX-A can suppress the expression of TGF-β1 in the TGF-β/small mother against decapentaplegic (Smad) pathway, effectively halting fibrosis in dermal tissues and cells. The studies indicated that rats and rabbits injected with BTX-A into the knee joint following knee joint trauma from postoperative immobilization showed reduced adhesion and joint fibrosis, resulting in improved movement limitation [9, 10].

Static progressive stretching (SPS) employs stress relaxation principles to manipulate soft tissues using adjustable braces. This enables precise control over the flexion and extension angles of joints, which facilitates continuous, gentle, and static stretching [11]. Therefore, this approach significantly improves ROM. A study demonstrated that treating traumatic knee flexion contracture in rats with SPS resulted in a significant reduction in the expression of TGF-β1 and IL-6. Additionally, there was a decrease in joint capsule adhesion and inflammation, inhibition of fibrosis and inflammatory changes in the joint capsule, and improvement in movement limitation [12].

This study aims to establish a rat model of knee stiffness by inducing joint fibrosis through post-traumatic flexion contracture of the knee. The hypothesis is that the combination of BTX-A and SPS could potentially prevent joint capsule fibrosis, leading to an improvement in joint stiffness.

## Materials and methods

### Animals

A total of 40 specific pathogen-free (SPF) adult male Sprague–Dawley (SD) rats, aged eight weeks and weighing between 220 and 300 g, were chosen from the Animal Experimental Center of Chengdu Sports University in Chengdu, China. The experimental rats were sourced from Chengdu Dashuo Experimental Animal Co. Ltd. (SCXK (Sichuan) 2020-0030).

### Sample size

The minimum sample size of six animals per group was calculated using G*power 3.1 software for animal sample size estimation [13]. With an estimated loss of 20%, the corrected sample size was determined to be eight animals per group, resulting in a total of 40 animals.

### Knee joint fibrosis stiffness modeling

The 40 rats were separated into two groups: a control group (group C, *n* ═ 8) and an experimental group consisting of 32 rats induced with knee joint stiffness. The rats were sedated using an intraperitoneal injection of a 1% solution of pentobarbital sodium (0.3 mL/100 g). The knee flexion stiffness model following trauma was built using the techniques developed by Hildebrand [14]. During the procedure, the muscle was incised to expose the lateral femoral condyle. An electric drill was used to create a hole 2 mm in width and 5 mm in depth. To simulate intra-articular fracture with blood flow into the joint cavity, care was taken to avoid damage to surrounding nerves and vessels. A Kirschner wire (0.8 mm) was inserted into the femur and tibia shafts. The knee joint was positioned at a maximum flexion of 150∘, and the excess part of the Kirschner wire was curved into a circular fixation using surgical forceps to avoid internal soft tissue injury after suturing. Finally, the wound was cleaned, disinfected, and closed.

### Animal groups

**Figure 1.**
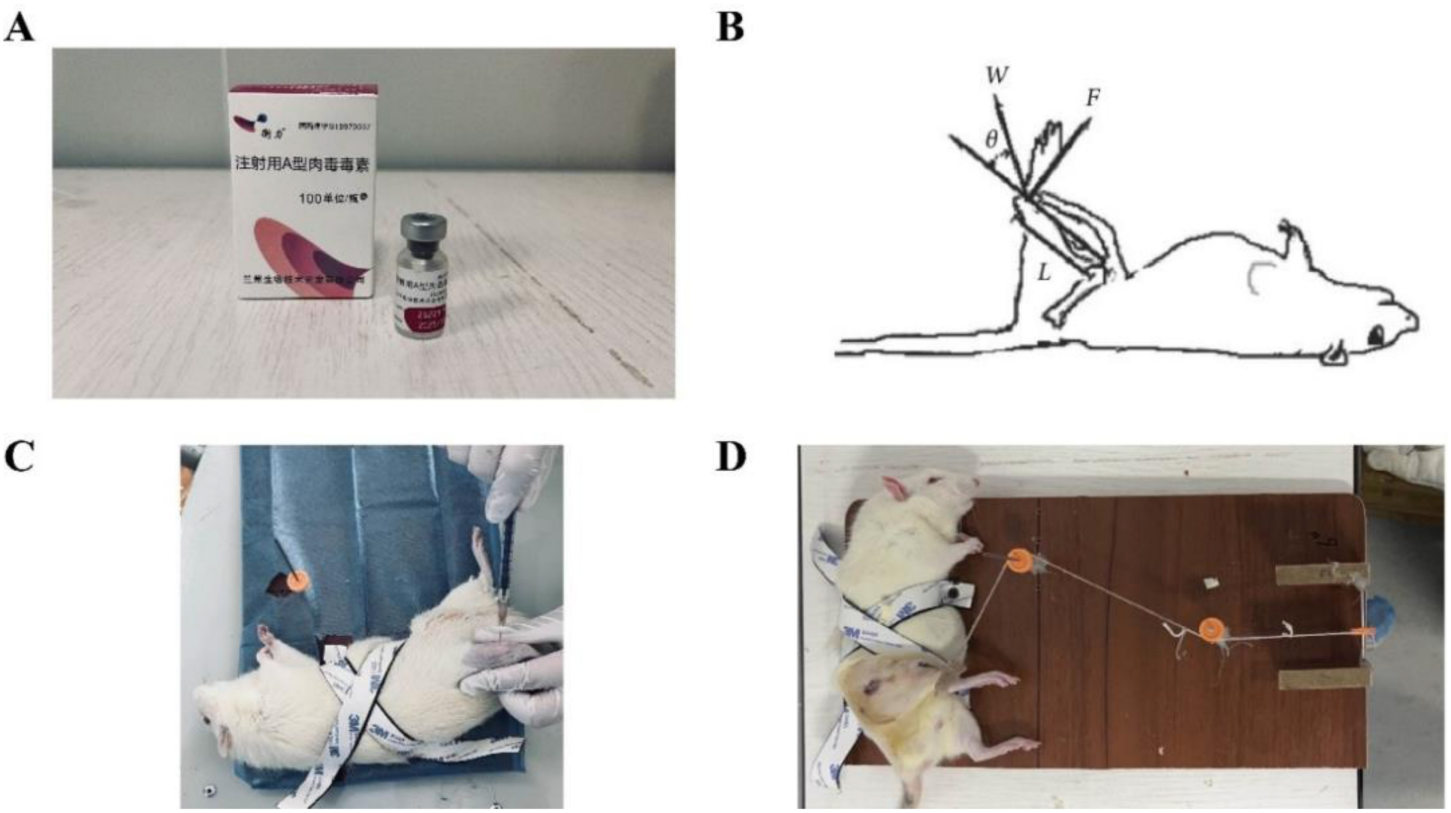
**BTX-A injection and SPS intervention.** (A) Botulinum toxin type A (BTX-A) used for experiments; (B) Static progressive stretching (SPS) schematic diagram; (C) BTX-A injection intervention; (D) SPS therapy.

The experimental animals were grouped using a table of random digits with IBM SPSS Statistics, Version 25.0 software. The animals were grouped in different proportions before and after modeling. Following the operation, one rat succumbed to infection, while two rats experienced fractures. One rat was excluded due to an excessive angle of joint immobilization. Three rats that did not meet the inclusion criteria were euthanized using an intraperitoneal injection of a 1% sodium pentobarbital solution (1.5 mL/100 g). After excluding these four rats, 28 rats that met the criteria were randomly divided into a modeling control group (MC group), an SPS intervention group (SI group), a botulinum toxin intervention group (BI group), and a combined intervention group (CI group), with seven rats in each group (Table 1, Tables S1 and S2, and Figure 1).

**Table 1:** Intervention methods and sample size of each group

**Group**	**Quantity**	**Modeling**	**SPS**	**BTX-A**
C	8	−	−	−
MC	7	+	−	−
SI	7	+	+	−
BI	7	+	−	+
CI	7	+	+	+

“+” means yes, “−“ means no. SPS: Static progressive drafting; BTX-A: Botulinum toxin type A; C: Blank control group; MC: Modeling control group; SI: SPS intervention group; BI: BTX-A intervention group; CI: SPS combined with BTX-A intervention group.

### Inclusion and exclusion criteria

**Figure 2.**
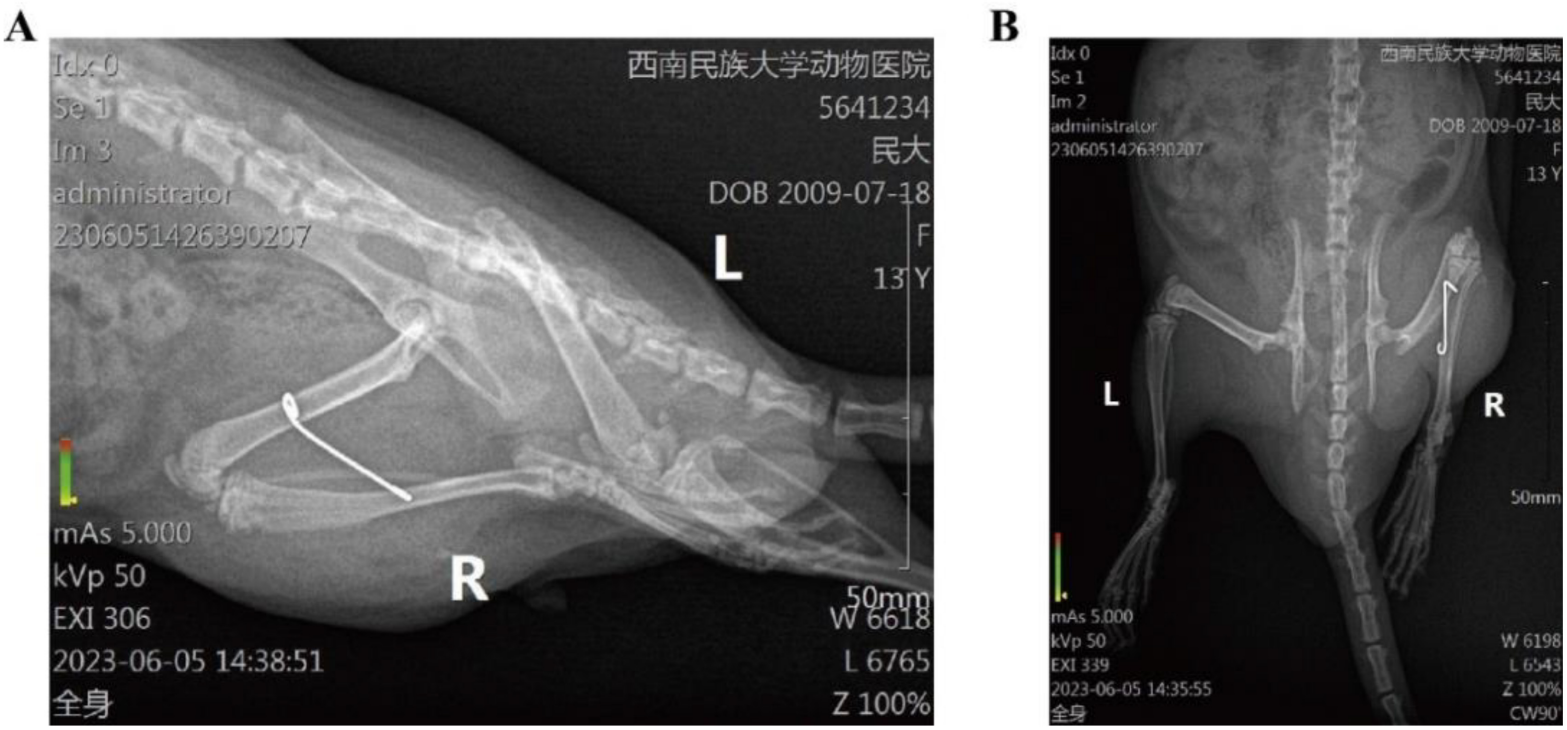
**X-rays of the rat knee joint.** (A) Lateral radiograph; (B) Anteroposterior radiograph.

(1) After modeling: ROM < 50∘ was considered successful. If ROM > 50∘, immobilization was deemed a failure, and those rats were excluded.

(2) X-ray: Rats were excluded if any of the conditions of knee joint fracture, dislocation, loosening, or breakage of the Kirschner wire occurred.

(3) After removal of immobilization: ROM < 70∘ indicated a successful model. If ROM > 70∘, immobilization was deemed a failure, and the rats were excluded (Figure 2).

### Static progressive stretching (SPS) intervention

The primary mode of movement for the knee joint is flexion or extension. Therefore, the knee joint can be described as a flexion and extension moment model. The SPS equipment is specifically designed and fabricated based on research conducted by Wang et al. [12], ensuring precise stretching accuracy. The tension force is regulated using pulleys, traction wires, and weights of 0.8 N.

**Figure 3.**
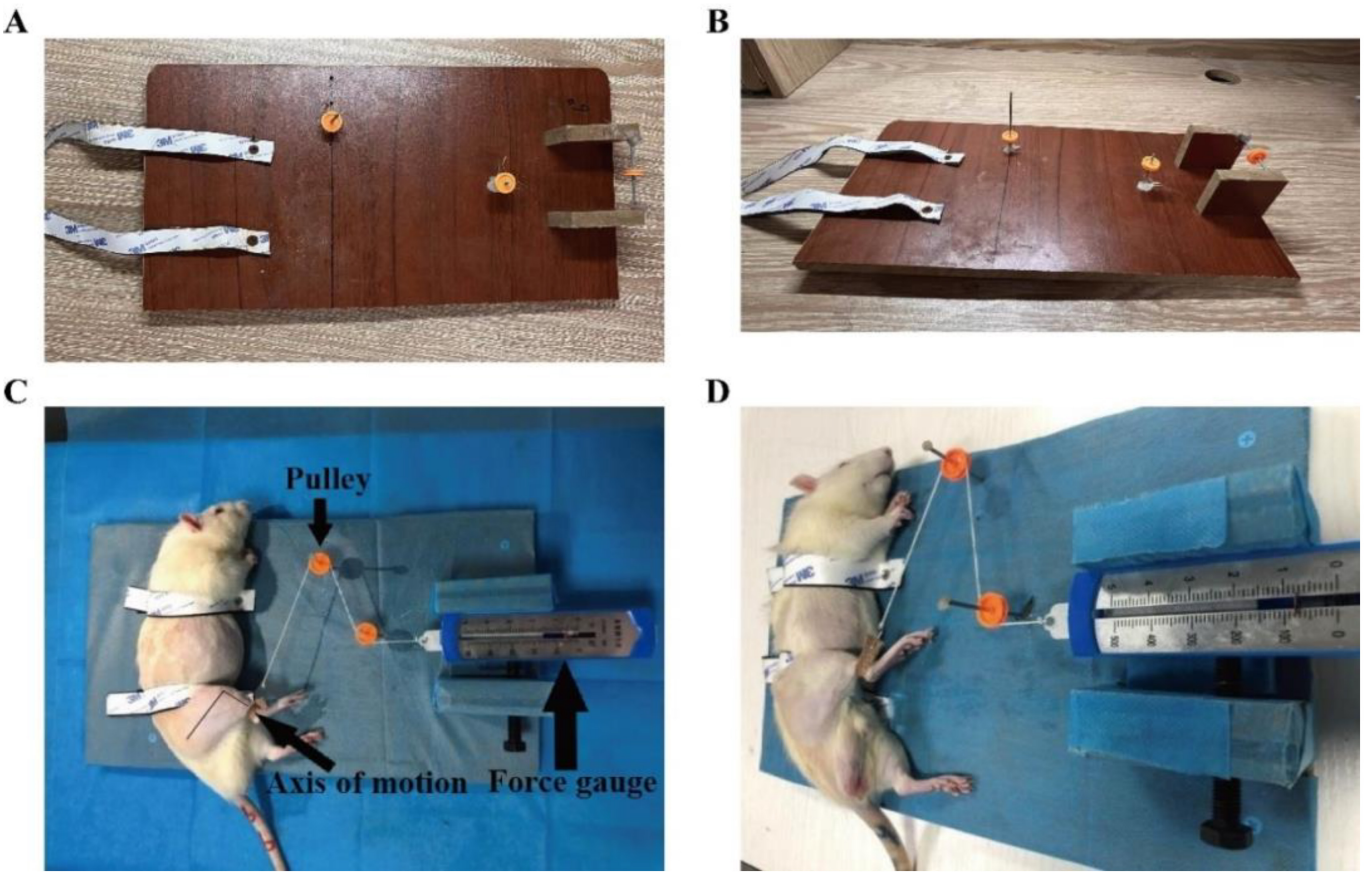
**Static progressive stretching (SPS).** (A) Vertical view of SPS equipment; (B) Side view of SPS equipment; (C) Vertical view of stretching rat; (D) Side view of stretching rat.

The knee extension moment was calculated by measuring the force exerted on the ankle joint. The SPS tensile strength was determined using the formula *T* ═ *F* ⋅ *L* ⋅ sinθ, where *T* represents the tensile torque measured in N⋅m and *F* represents the tensile force measured in Newtons (N). *L* represents the length of the rat tibia in meters (m), whereas θ represents the angle between the direction of stretching and the tibia. Assuming θ is set to 90∘, the stretching force is applied perpendicular to the rat tibia. The torque was set to *T* ═ 0.04 N⋅m, the length was *L* ═ 0.05 m, the sine of the angle was 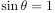, and the tension meter reading was calculated as *F* ═ 0.8 N. The traction force site was at the ankle joint of rats. Rats in the SI and CI groups were subjected to anesthesia and stretched using a 1% solution of sodium pentobarbital (0.3 mL/100 g) (Figure 3). The duration of the stretching intervention was 30 min each day for a total of 16 days.

### BTX-A intervention

Following a 4-week period of immobilization, a botulinum toxin injection was administered immediately after the internal immobilization was removed under anesthesia. The rats’ right knee was flexed at a 45∘ angle, with the puncture point located at the outer edge of the white patellar tendon, specifically at the lower pole of the patella. The needle was inserted from the intercondylar fossa and withdrawn 2 mm after reaching the femoral condyle. A solution of 100 units of BTX-A (Hengli, Lanzhou Biological Products Institute, Lanzhou, China) was mixed with 1 mL of 0.9% saline. From this mixture, 0.1 mL (10 units) of BTX-A solution was extracted using a 1 mL syringe and injected into the joint cavity [9]. The rats in the injection intervention groups received only one injection during the study.

### Tissue preparation

The tissue sampling took place 16 days after the removal of the fixation, marking the completion of the intervention. Before sampling, the rats in each group were weighed and then received a 1% intraperitoneal injection of pentobarbital sodium solution at a dosage of 0.5 mL per 100 g. After anesthesia, the ROM of the right knee joint was measured. Following sacrifice, the skin was peeled off and the stiffness of the joint was measured. The knee joint was surgically removed using forceps, with cuts made 1–2 cm above and below the joint. Following the removal of the complete knee joint and trimming of the surrounding muscles, the collected specimens were divided for pathologic staining. The samples were then stored in 10% neutral formaldehyde and a −80 ^∘^C refrigerator, respectively.

### The measurement of knee joint ROM

The ROM of the right knee joint was measured in all rats both before and after modeling, as well as before and after intervention. The measurement was conducted under anesthesia, with rats positioned on a knee joint angle measuring device developed in-house (Patent: No. 202221015930.0). Based on the selection of *F* ═ 0.7 N, this moment represents the minimum safe amount of force required to stretch the knee joint to its maximum angle without causing damage to the knee joint capsule [15]. The measurement is taken with the intersection of the long axis of the femur and the extended axis of the tibia as the center point. The fixed arm represents the long axis of the femur, while the moving arm represents the long axis of the tibia. The pulling force is applied from the center of the lateral ankle, in a direction perpendicular to the long axis of the tibia. The average value is obtained by repeating the measurement three times.

### Joint stiffness calculation

Joint stiffness is defined as the ratio of the change in knee joint torque to the change in knee joint ROM within the maximum ROM (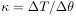, where 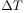 is the change in knee joint torque, and 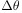 is the change in knee joint motion angle). Based on the knee joint stiffness measurement standard developed by Zhou et al. [16], torques of 2.5, 7.5, 12.5, and 17.5 N⋅cm were chosen. These torques correspond to forces of 0.5, 1.5, 2.5, and 3.5 N, respectively. These values were used to compute joint stiffness. The difference between the ROM measured under various tensile forces and the ROM at a 45∘ angle was calculated. Following three repetitions of the measurement, the average value was determined to evaluate the degree of joint stiffness in rats. The measurement method used was identical to that of ROM.

### H&E and Masson staining

After the knee joint samples were fully fixed using a 10% neutral formaldehyde solution for at least 24 h, the tissues were extracted and immersed in a 15% EDTA decalcification solution. The decalcification solution was substituted every three days until the decalcification process was completed after eight weeks. The tissue was dehydrated using a dehydrator and then embedded in paraffin. The samples were sliced into 5 µm sections using a slicer. Following the removal of paraffin, the samples were stained with H&E and Masson as per the provided instructions. This allowed for the identification of pathological changes and collagen deposition in the joint capsule. The digital slice scanner 3DHISTECH (Pannoramic 250, Hungary) was used to collect images. All tissues were observed at low magnification first, and then images at ×400 magnification were collected in the same area of each sample. A four-grade scoring scale [17, 18] was employed to assess the pathogenic and cell morphological alterations in synovial tissue. The total number of cells and collagen deposition were analyzed using Image Pro Plus 6.0 software. The total number of cells was expressed as cells per square millimeter of joint capsule area, and collagen deposition was expressed as a percentage of blue area (Table S3).

### Western blot protein detection

The articular capsule tissue samples were pulverized using a grinder, then homogenized and centrifuged in RIPA lysate (Biosharp, China). The liquid fraction obtained after this process was referred to as the supernatant. Protein quantification was conducted using the BCA protein assay (BiTianbio, China). Subsequently, an equal quantity of protein from each pair of samples was isolated using SDS-PAGE on Bis-Tris 4%–20% high-resolution precast gels and transferred onto polyvinylidene fluoride microporous membranes via electrotransfer. The membranes were then dissolved in TBST after being blocked with 5% skim milk and incubated at room temperature for 2 h. The membranes were incubated with rabbit anti-Smad2 (1:1000;abclonal), rabbit anti-Smad3 (1:2000;abclonal), rabbit anti-TGF-β1 (1:1000;abclonal), rabbit anti-p-Smad2 (1:1000;abclonal), rabbit anti-p-Smad3 (1:2000;abclonal), rabbit anti-Smad4 (1:2000;abclonal), rabbit anti-Collagen I (1:1000;abclonal), rabbit anti-Collagen III (1:1000;abclonal), rabbit anti-α-SMA (1:1000;abclonal), and β-actin (1:50000;abclonal) at 4 ^∘^C overnight. On the second day, the membranes were washed three times for 10 min each with TBST solution. Then, they were incubated with a secondary antibody, specifically goat anti-rabbit IgG (H+L) HRP (1:5000; affbiotech), for 45 min at room temperature. After that, the membranes were washed three more times for 10 min each with TBST solution. Finally, ECL chemiluminescent droplets were added to the target bands, and the signal was detected with digital imaging equipment. The densities of each band were quantified using the Tanon fluorescence image analysis system software V2.0. The bands were exposed, and Gel-pro analyzer4 scanned the exposure results and expressed them as the integrated optical density (IOD) of the target proteins. The protein expression levels of TGF-β1/Smad were calculated by comparison with the amount of β-actin as a loading control.

### Ethical statement

The authors ensured that animal care adhered to the guidelines set by their institution and the Ministry of Science and Technology of Chinese Guidance Suggestions for the Care and Use of Laboratory Animals. The animal tests were conducted in accordance with the ethical criteria for experimental animals as specified in the Chengdu Institute of Physical Education Ethics [2023] No. 43. The surgical procedures were conducted using sodium pentobarbital anesthesia, with utmost care taken to minimize any potential discomfort.

### Statistical analysis

The statistical analysis in this experiment was conducted using IBM SPSS Statistics, Version 25.0 software. The data consists of measurement data, specifically described by the mean and standard deviation (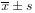). Following the Shapiro–Wilk and Levene tests, it was concluded that the data adheres to a normal distribution and exhibits homogeneity of variance. A paired sample *t*-test was utilized to compare the ROM of the knee in rats within each group, both before and after intervention, as well as before and after modeling. Following the intervention, the ROM, joint stiffness, and western blot indexes were analyzed using single component analysis of variance. Post-event comparisons were made using the least significant difference (LSD) approach. Differences were considered statistically significant when the *P* value was less than 0.05.

## Results

### ROM

Except for group C, there was no significant difference in knee joint immobilization angle among the groups before modeling (*P* > 0.05). Prior to the treatment, the rats exhibited an average ROM of less than 70, indicating the successful establishment of the model. After the intervention, there was a significant difference in ROM among the groups (*P* < 0.05). ROM was compared between rats in each group before and after the intervention. The ROM of rats in each group, except for group C, increased significantly compared to before intervention (*P* < 0.05). There was no significant difference in improving ROM between the SI group and the BI group (*P* > 0.05). The increase in ROM in the CI group was more pronounced compared to the SI and BI groups, showing a significant difference (*P* < 0.05) (Tables 2 and 3).

**Table 2:** Comparison of ROM before and after rat modeling in each group

**Group**	**Before Modeling ROM (∘)**	**After Modeling ROM (∘)**	**t**	***P***
C	124.85±2.65	126.24±2.60^b^	−8.342	0.360
MC	125.26±2.46	46.88±1.20^a^	77.223	<0.0001
SI	125.44±2.406	47.16±2.09^a^	88.398	<0.0001
BI	126.38±2.546	46.51±1.84^a^	70.252	<0.0001
CI	124.16±3.16	46.83±2.18^a^	52.094	<0.0001
F	0.752	2439.406		
*P*	0.563	<0.0001		

Compared with before modeling, ^a^*P* < 0.05; Compared with MC group, ^b^*P* < 0.05. ROM: Range of motion; SPS: Static progressive drafting; BTX-A: Botulinum toxin type A; C: Blank control group; MC: Modeling control group; SI: SPS intervention group; BI: BTX-A intervention group; CI: SPS combined with BTX-A intervention group.

**Table 3:** Comparison of ROM before and after rat intervention in each group

**Group**	**Before Intervention ROM**	**After Intervention ROM**	**t**	***P***
C	125.73±2.40	124.87±3.00	1.071	0.320
MC	65.98±4.70	86.95±3.54^acde^	−20.360	<0.0001
SI	66.51±3.97	111.71±3.73^abe^	−17.669	<0.0001
BI	63.69±4.81	113.25±4.15^abe^	−29.464	<0.0001
CI	63.12±5.17	117.59±1.73^abcd^	−0.980	<0.0001
F	287.612	126.889		
*P*	0.000	<0.0001		

Compared with before intervention, ^a^*P* < 0.05; Compared with MC group, ^b^*P* < 0.05; Compared with SI group, ^c^*P* < 0.05; Compared with BI group, ^d^*P* < 0.05; Compared with CI group, ^e^*P* < 0.05. ROM: Range of motion; SPS: Static progressive drafting; BTX-A: Botulinum toxin type A; C: Blank control group; MC: Modeling control group; SI: SPS intervention group; BI: BTX-A intervention group; CI: SPS combined with BTX-A intervention group.

### Joint stiffness

**Figure 4.**
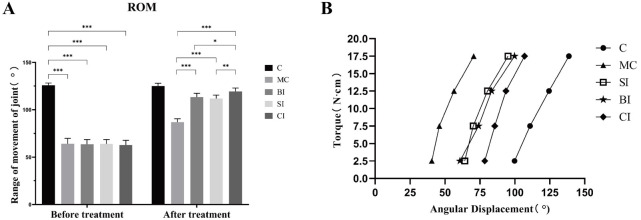
**ROM and joint stiffness comparison of rats.** (A) ROM of rats before and after treatment; (B) Joint stiffness of rats knee. ROM: Range of motion; SPS: Static progressive drafting; BTX-A: Botulinum toxin type A; C: Blank control group; MC: Modeling control group; SI: SPS intervention group; BI: BTX-A intervention group; CI: SPS combined with BTX-A intervention group. *: *P* < 0.05; **: *P* < 0.01; ***: *P* < 0.001; ****: *P* < 0.0001.

After treatment, angular displacement under different torque was observed between groups (Figure 4). In group C, the knee joint stiffness, which without modeling, exhibited a consistent linear alteration. Specifically, the change in angular displacement per unit torque remained nearly constant. In the MC group, the largest slope ratio indicated that knee joint stiffness was the greatest, making it the stiffest. The slope in the BI group was similar to that of the SI group, showing that the stiffness difference between these two groups was not significant. Therefore, comparing the efficacy of BTX-A and the mechanical effect of SPS requires a more detailed analysis of different doses. Compared to the other groups, the CI group showed a more significant change, with a notably lower slope, indicating that the combined intervention was most effective in improving joint stiffness and significantly reducing the degree of stiffness (Figure 4).

### Histological change

**Figure 5.**
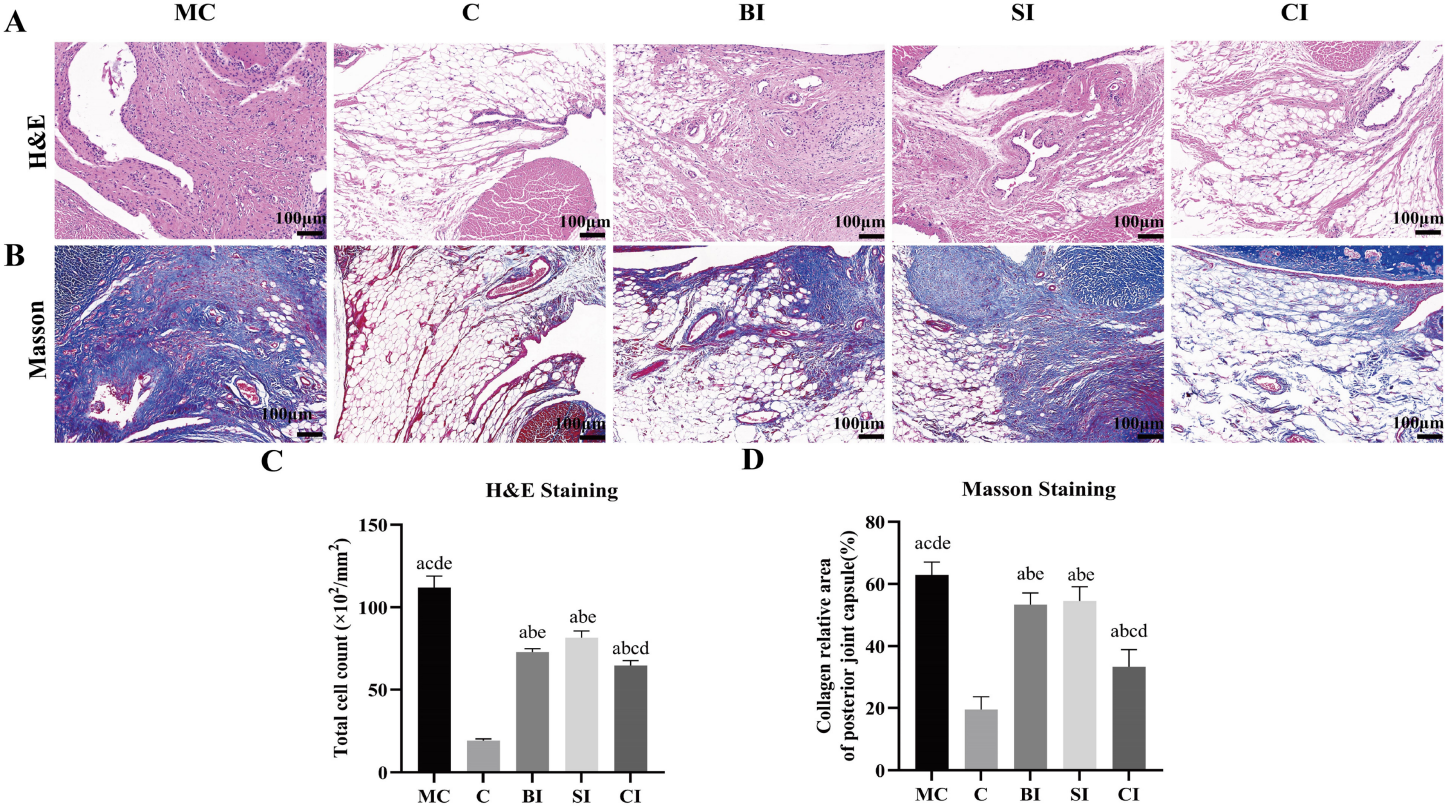
**H&E staining and** **Masson** **staining.** (A) Results of H&E staining; (B) Results of Masson staining; (C) Quantitative analysis of the number of total cells in each group; (D) Quantitative analysis of the percentage of collagen deposition (blue area) in each group. Data are expressed as mean ± standard deviation. ^a^*P* < 0.05 vs the C group, ^b^*P* < 0.05 vs the MC group, ^c^*P* < 0.05 vs the BI group, ^d^*P* < 0.05 vs the SI group, ^e^*P* < 0.05 vs the CI group. Scale bars ═ 100 µm. C: Blank control group; MC: Modeling control group; SI: SPS intervention group; BI: BTX-A intervention group; CI: SPS combined with BTX-A intervention group.

The results showed that in group C, the synovial tissue structure was clear and complete, with its lining layer composed of single-layer or double-layer synovial cells, which were loosely arranged and partially discontinuous. The lower layer of the lining consisted of loose connective tissue, primarily composed of fat cells, fibroblasts, and blood vessels. The synovial membrane showed clear signs of damage, with fibrous tissues replacing the original synovial tissue. Adipose tissue was mostly absent, and there was dense hyperplastic collagen, along with increased blood vessels and a small amount of inflammatory cell infiltration, specifically lymphocytes. Observations revealed slight fibrous tissue hyperplasia, accompanied by a small number of blood vessels or lymphocytes, in both the BI group and SI group. In the CI group, a distinct sliding membrane structure was observed with a slight increase in synovial cells in the lining layer. No significant fibrous tissue hyperplasia or inflammatory cell infiltration was detected in the lower lining layer (Tables 4 and 5 and Figure 5).

**Table 4:** Pathological changes in synovial tissue by H&E staining

**Group**	**Result**
C	Tissue cell boundaries clear, neat rows, no obvious pathological changes (−)
MC	Synovial hyperplasia of fibrous tissue (+++) Increased blood vessels (++) Inflammatory cell infiltration (+)
BI	Synovial hyperplasia of fibrous tissue (++) Increased blood vessels (+)
SI	Synovial hyperplasia of fibrous tissue (+) Inflammatory cell infiltration (+)
CI	Increased number of synovila cells (+)

Normal (−), Minor (+), Mild (++), Moderate (+++), Severe (++++). SPS: static progressive drafting; BTX-A: Botulinum toxin type A; C: Blank control group; MC: Modeling control group; SI: SPS intervention group; BI: BTX-A intervention group; CI: SPS combined with BTX-A intervention group.

**Table 5:** Collagen deposition in synovial tissue by Masson staining

**Group**	**Result**
C	The synovial tissue structure was complete and clear, with no obvious fibrous tissue proliferation (−)
MC	Significant proliferation of synovial fibrous tissue (++++)
BI	A small amount of hyperplasia of synovial fibrous tissue (++)
SI	Moderate hyperplasia of synovial fibrous tissue (+++)
CI	Minimal hyperplasia of synovial fibrous tissue (+)

Normal (−), Minor (+), Mild (++), Moderate (+++), Severe (++++). SPS: Static progressive drafting; BTX-A: Botulinum toxin type A; C: Blank control group; MC: Modeling control group; SI: SPS intervention group; BI: BTX-A intervention group; CI: SPS combined with BTX-A intervention group.

### Western blot protein detection

**Figure 6.**
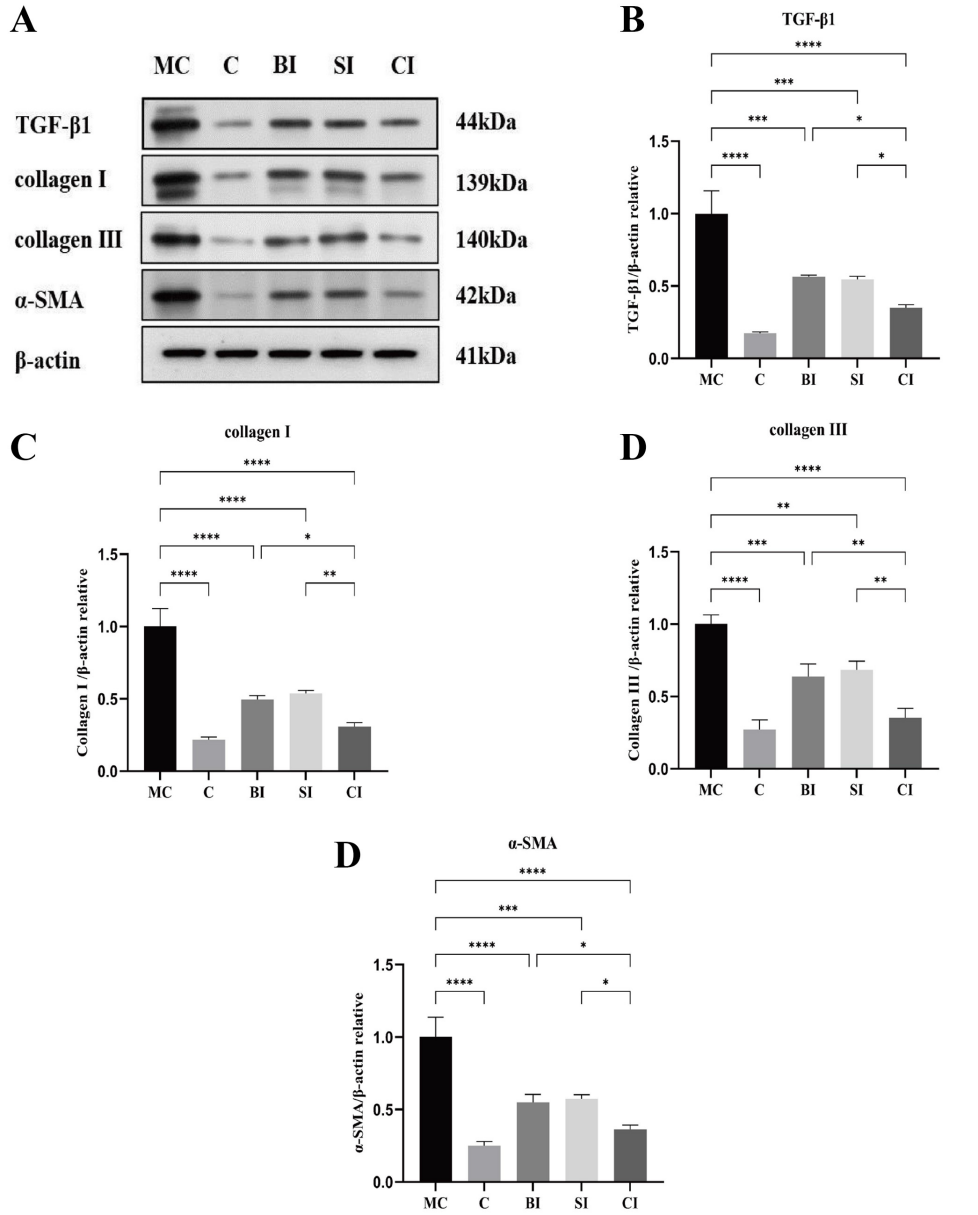
**Protein expression levels of TGF-β1, collagen I, collagen III, and α-SMA relative to β-actin.** (A) Western blotting bands of TGF-β1, collagen I, collagen III, α-SMA and β-actin proteins; (B) Quantitative analysis of TGF-β1; (C) The quantitative analysis of collagen I; (D) Quantitative analysis of collagen III; (E) Quantitative analysis of α-SMA. Data are expressed as mean ± standard deviation. C: Blank control group; MC: Modeling control group; BI: BTX-A intervention group; SI: SPS intervention group; CI: SPS combined with BTX-A intervention group; TGF-β1: Transforming growth factor beta-1; Collagen I: Collagen type I; Collagen III: Collagen type III; α-SMA: Alpha-smooth muscle actin; β-actin: Beta-actin. *: *P* < 0.05; **: *P* < 0.01; ***: *P* < 0.001; ****: *P* < 0.0001.

The relationship between the average protein expression levels of TGF-β1, Collagen I, Collagen III, α-SMA, and β-actin in the five groups of specimens is shown in Figure 6A.

The protein expression levels of TGF-β1, collagen I, collagen III, and α-SMA in the BI, SI, and CI groups were significantly lower than those in the MC group (*P* < 0.05). The protein expression levels of TGF-β1, collagen I, collagen III, and α-SMA in the CI group were lower than those in the BI group (*P* < 0.05) and SI group (*P* < 0.05), with no significant difference between the BI group and SI group (*P* > 0.05). The protein expression levels of TGF-β1, collagen I, collagen III, and α-SMA in the CI group were not significantly different from those in the C group (*P* > 0.05) (Figure 6).

**Figure 7.**
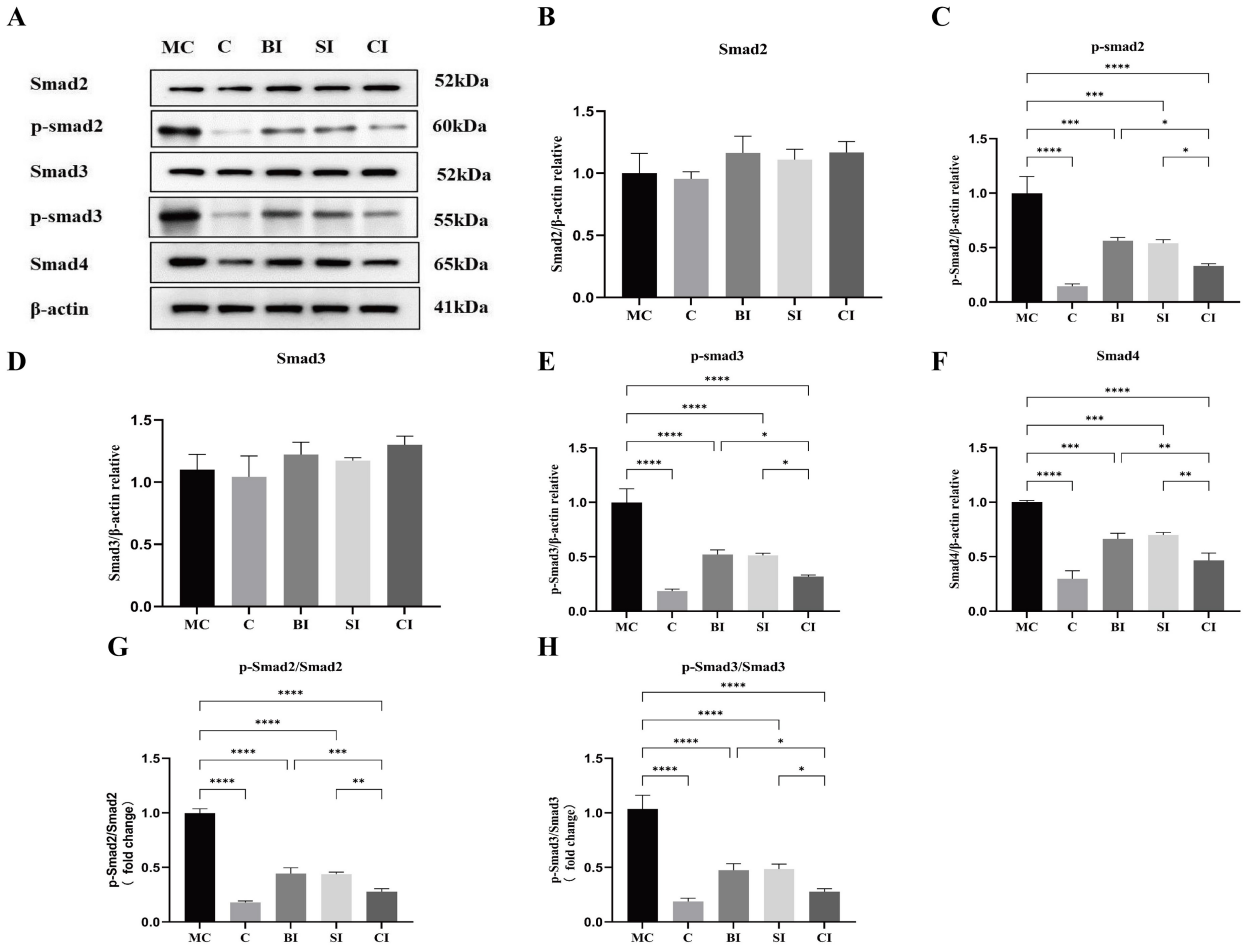
**Protein expression levels of** **Smad2, Smad3, Smad4, and p-Smad2∖3 relative to β-actin.** (A) Western blotting bands of Smad2, Smad3, Smad4, and p-Smad2∖3 and β-actin proteins; (B) Quantitative analysis of Smad2; (C) Quantitative analysis of p-Smad2; (D) Quantitative analysis of Smad3; (E) Quantitative analysis of p-Smad3; (F) Quantitative analysis of Smad4; (G) Ratio analysis of p-Smad2/Smad2; (H) Ratio analysis of p-Smad3/Smad3. Data are expressed as mean ± standard deviation. C: Blank control group; MC: Modeling control group; BI: BTX-A intervention group; SI: SPS intervention group; CI: SPS combined with BTX-A intervention group. Smad2: Small mother against decapentaplegic 2; Smad3: Small mother against decapentaplegic 3; Smad4: Small mother against decapentaplegic 4; p-Smad2: Phosphorylated-Smad2; p-Smad3: Phosphorylated-Smad3; β-actin: Beta-actin. *: *P* < 0.05; **: *P* < 0.01; ***: *P* < 0.001; ****: *P* < 0.0001.

The relationship between the average protein expression levels of Smad2, Smad3, Smad4, p-Smad2∖3, and β-actin in the five groups of specimens is shown in Figure 7A. The results showed that compared with group C, there was no significant difference in the protein expression levels of Smad2 and Smad3 in the modeled groups (*P* > 0.05), while the protein expression levels of TGF-β1, p-Smad2∖3, and Smad4 in the modeled groups were significantly increased (*P* < 0.05), with the MC group being the most significant (*P* < 0.01). The phosphorylation level can be shown by the ratio of phosphorylated protein to total protein. The phosphorylation levels of Smad2 and Smad3 in each group of rats were significantly higher compared to group C, with an incredibly significant difference (*P* < 0.01). There was no significant difference between the BI group and SI group (*P* > 0.05). Compared with the BI group and SI group, the phosphorylation level in the CI group decreased significantly (*P* < 0.05) (Figure 7).

## Discussion

### Effect on the function of stiff knee joint in rats

#### Effect on ROM

Activity limitation is a notable sign of joint stiffness, and its severity gradually increases over time. ROM is an essential metric used to evaluate the extent of joint stiffness. The study’s results suggest that except for group C, the ROM in rats from the remaining groups was limited following the modeling procedure. After four weeks, when the immobilization was removed, the rats in each group experienced some relief from joint activity limitation. However, it was observed that the joint activity limitation did not fully return to the same level as that in group C, suggesting that it was not completely reversible. The improvement in ROM in the MC group is not satisfactory compared to other intervention groups, indicating that the intervention has a better effect than natural recovery, which aligns with previous findings [19]. This could be due to the inadequate control of inflammation during the active phase after prolonged immobilization, leading to increased fibrosis and stiffness.

When comparing the recovery of ROM between the BI group and SI group, no significant difference was observed among the various intervention groups. There is a scarcity of pertinent comparison studies on the efficacy of curative effects. This study, along with earlier studies [12, 20], examines the rationale at the action principal level of the two therapies. At the primary level of the two interventions: Stress relaxation on joints with SPS enhances the ROM; however, this physiological enhancement is temporary. The study demonstrates that BTX-A can decrease the development of intra-articular adhesion by enhancing joint inflammation through its interaction with nociceptors and inhibition of pain-related variables such as substance P and glutamate release [21]. Nevertheless, there is currently no definitive suggestion for the ideal dosage for injections in relevant investigations. At the intervention, we observed a rapid change in the ROM of rats in the BI group at the early stage. One week after injection, there was a noticeable improvement in ROM. However, the early recovery progress of rats in the SI group was not as favorable as that in the BI group. After analyzing the reasons, we speculated that it might be due to the diffusion of botulinum toxin [22]. Although BTX-A was injected into the joint cavity to avoid the direct effect of intramuscular injection on muscles, it was still inevitable that the drug diffusion invaded the muscles and soft tissues around the knee joint, resulting in decreased of muscle tension. However, whether the diffusion of BTX-A affects joint function needs more experiments. In addition, we found that the improvement of ROM in the CI group was more obvious than that in the BI and SI groups, indicating that combined intervention was better than single intervention. On this basis, we can explore more suitable drug dosages and stretching strength to improve the treatment effect.

#### Effect on joint stiffness

Trudel et al. [3] demonstrated that short-term immobilization leads to joint stiffness mostly caused by muscle-derived factors, whereas long-term immobilization results in joint stiffness primarily caused by joint-derived factors. The evaluation of knee joint performance in clinical settings relies on two important indices: joint stiffness and ROM. These indices measure distinct biomechanical features of the joint [16]. Among them, the ROM of the knee joint is a method of evaluating the angle limitation when a single torque value is applied. On the other hand, joint stiffness measures the resistance generated by passive joint activities in response to different torques. It is expressed as the change in unit torque divided by the angular displacement caused by knee joint activities. In Figure 4B, joint stiffness is represented by the slope, where a smaller slope indicates a greater change in angular displacement under unit torque, indicating higher stiffness.

Our research findings indicate that the joint stiffness in group C exhibits a consistent linear relationship at various torque levels, and there is no correlation between torque increase and joint stiffness rise or reduction. However, in comparison to Group C, the other groups have experienced varying degrees of increased joint stiffness, suggesting the presence of a certain level of joint stiffness in the immobilized knee joint. The most prominent rise in stiffness is observed in the MC group, suggesting that joint stiffness continues to develop even after the restricted joint is allowed to move again, compared to its stiffness prior to immobilization. Prior research has indicated that the stiffness of the knee joint does not exhibit any significant statistical differences within a period of four weeks following simple immobilization [16]. Furthermore, there is no discernible variation until eight weeks. Additionally, the alteration in joint stiffness is associated with both the duration of immobilization and the time taken for reactivation. If the immobilization period exceeds four weeks, even if the reactivation duration is extended, the rigid joint’s stiffness improvement cannot be fully regained to its pre-immobilization state.

This study required the model to be immobilized for a duration of four weeks following the trauma. The results showed a notable rise in rigidity, which can be ascribed to the recuperation process linked to the injury. Based on the slope *k* of each group, the overall change trend of the BI group and SI group is similar. This means that there is only a slight difference in stiffness between them under different torques. This indicates that both therapies may effectively improve joint stiffness. SPS may induce continuous stress relaxation in rigid joints from a biomechanical perspective and also enhance the degradation of connective tissue media [23], hence improving joint stiffness. BTX-A can decrease the quantity of fibroblasts in fibrotic connective tissue. A previous study [9] discovered that the injection of BTX-A into the knee joint of rats can decrease the quantity of fibroblasts in the cartilage and alleviate joint stiffness resulting from adhesion. Furthermore, we observed a substantial decrease in the stiffness of the CI group. This could be attributed to the synergistic impact of the two interventions, resulting in a more pronounced therapeutic effect. These findings suggest that the combined treatment has a superior effect in decreasing joint stiffness.

### Effect on physiological effects of stiff knee joint in rats

#### Effect on histology

Following knee joint trauma, the accumulation of collagen and the inflammatory response in the joint are key factors in promoting the development of joint capsule fibrosis. This is particularly true after prolonged immobilization, which can result in injury to the joint capsule and a persistent inflammatory response [24]. The presence of specialized fibroblasts and inflammatory cells in the affected tissue contributes to long-term stiffness. Hence, when examining the pathological alterations of rigid joints, it is crucial to focus on the cells associated with inflammation and fibrosis.

Pathological changes can be observed when examining knee joints using a light microscope. The changes observed consist of an augmentation in the growth of fibrous tissue, the development of new blood vessels, the infiltration of inflammatory cells, and a slight elevation in the quantity of synovial cells. These modifications are linked to the ongoing experimental therapy. Based on the findings from H&E and Masson staining, we observed no pathological alterations or inflammatory responses in group C. However, in comparison to group C, the other groups exhibited varying degrees of fibrous tissue hyperplasia, along with an increase in blood vessels or lymphocytes. Among all the groups, the MC group shows the most severe pathological changes. This suggests that joint fibrosis will continue to progress and both fibrosis and the inflammatory reaction will persist after immobilization. These findings align with previous results [16, 25]. The intervention groups showed a significant decrease in the proliferation of fibrous tissue and lymphocytes compared to the MC group. This suggests that the intervention effectively controlled and improved fibrosis and inflammatory reactions to some extent. In comparison to the SI group, the BI group exhibits a relatively smaller difference in the degree of fibrous tissue proliferation. Additionally, the SI group has a higher number of lymphocytes compared to the BI group, which could be attributed to the anti-inflammatory effect of BTX-A. On the other hand, when compared to both the BI and SI groups, the CI group demonstrates the lowest level of fibrous tissue proliferation. However, there is no significant increase in the number of blood vessels and lymphocytes in the CI group. These results show that both SPS and BTX-A can improve fibrosis, and the combined use of SPS and BTX-A has a more significant inhibitory effect on tissue fibrosis.

The infrapatellar fat pad (IFP) is a structure located within the knee joint, outside of the synovial membrane. It is a triangular adipose tissue mass situated in the intervertebral space of the patella, the anterior inferior aspect of the femoral condyle, the anterior superior border of the tibia, and the posterior region of the patellar ligament, which occupies the front part of the knee and has a high blood supply and nerve supply. Its presence contributes to the stability of the knee joint and helps to minimize friction [26]. When the IFP experiences inflammation and fibrosis due to trauma or surgery, it can result in various arthrofibrotic lesions. The study demonstrates that BTX-A could enhance the engraftment of adipose tissue by stimulating cell proliferation, adipogenesis, and angiogenesis [27]. In addition, stretching exercises are frequently used to treat IFP disease. The study demonstrates that the size and movement of the IFP are altered by manual release or stretching during quasi-static knee extension in patients with knee osteoarthritis, leading to an improvement in knee ROM [28]. The results of H&E staining revealed a significant reduction in adipose tissue and a significant increase in fibrous tissue in the MC group. However, the intervention group showed varying degrees of improvement in fibrosis, particularly in the CI group.

#### Effect on TGF-**β**1/Smad signal pathway

Studies have shown that activated TGF-β1 facilitates the binding of phosphorylated Smad2 and Smad3 with Smad4, thereby regulating the production of collagen, fibronectin, and protein–polysaccharide by fibroblasts in the nucleus [25, 29]. This process leads to the deposition of ECM and the transformation of EMT, resulting in the formation of fibrosis [6, 30]. Consequently, in the TGF-β1/Smad pathway, TGF-β1, Smad2, and Smad3 are pivotal proteins for activating the pathway. Regulating the expression and/or phosphorylation of these factors can inhibit fibrosis and decrease the formation of joint capsule fibrosis, thereby reducing the transformation efficiency of fibrosis. Previous research has verified that α-SMA, collagen I, and collagen III are crucial constituents of collagen accumulation and have a substantial impact on the development of joint capsule fibrosis [31, 32].

Similarly, the results of this experiment show that the phosphorylation levels of TGF-β1, Smad2, Smad3, and Smad4 in the knee joint capsule tissue in the MC group are significantly higher than those in the C group, suggesting that the activation of TGF-β1/Smad pathway is one of the molecular mechanisms involved in the formation of knee joint capsule fibrosis. Among them, the contents of α-SMA and collagen I and III in the MC group also increased significantly, indicating that reactivation after long-term immobilization would aggravate collagen deposition in joints and promote the progression of fibrosis, which is consistent with previous research results [19].

Extensive research has been conducted on the impact of SPS and BTX-A on fibrosis. After applying SPS intervention to the knee joint of inflexible rats, Zhang et al. [33] observed a significant reduction in the levels of TGF-β1 and collagen accumulation in the joint capsule of the affected joint, in comparison to the model group. In addition, Wang et al. [12] verified that a solitary 30-min SPS treatment yielded the highest efficacy. Furthermore, in Kim’s study [6], it was discovered that the addition of TGF-β1 and BTX-A to human fibroblasts, either separately or together, resulted in the inhibition of TGF-β1 fibroblast differentiation. This inhibition led to the suppression of type I and type III collagen synthesis and a decrease in p-Smad2∖3 levels. These findings provide evidence for the anti-fibrosis properties of BTX-A on contracture tissues.

Out of all the groups that intervened, the CI group had the lowest protein expression level, indicating that combining interventions may be more beneficial than using a single intervention. Both SPS and BTX-A can suppress the expression of the TGF-β/Smad pathway. When used together, they have a more potent inhibitory effect on this pathway, which significantly delays the progression of joint capsule fibrosis.

### Shortcomings and prospects

There are certain constraints in this experiment. The duration of this experiment is fixed at four weeks, and there is no comparison between different cycles of immobilization. This aspect could be included in future studies. The injection dose concentration of the BI group and the drafting strength and frequency of the SI group were determined based on previous relevant experiments [9, 12]. However, the effects of varying doses, drafting strength and frequency, and different time periods have not been confirmed. Furthermore, at present, only ROM and joint stiffness are chosen as functional indicators. In the future, additional indicators, such as muscle cross-sectional area, muscle strength, gait, pain, and proprioception may be considered.

Our future research will prioritize improving the experimental methodology and examining the effects of different doses and durations on knee joint fibrosis and stiffness. Additionally, we will integrate supplementary significant indicators and engage in further investigation regarding cytokines, protein genes, and pathways associated with regulation and other pertinent systems.

## Conclusion

The findings of our study indicate that the activation of the TGF-β/Smad pathway is a contributing factor in the progression of joint capsule fibrosis leading to joint stiffness. SPS and BTX-A can decrease the phosphorylation levels of Smad2 and Smad3 by inhibiting the excessively activated TGF-β1 in the joint capsule. This prevents them from binding with the Smad4 receptor, which in turn affects the expression of downstream proteins, including collagen I, collagen III, and α-SMA. As a result, this improves joint stiffness caused by fibrosis of the joint capsule.

Hence, we posit that the combination of SPS and BTX-A is efficacious in addressing knee joint fibrosis. This finding warrants additional investigation and refinement, as it holds promise and importance for wider adoption.

## Supplemental data

**Table S1:** Random number table before modeling

**Weight (g)**	**Serial number**	**Random numbers**	**Group number**
220.21	1	9.32	2
221.32	2	8.65	2
224.00	3	9.99	2
227.03	4	3.67	1
227.20	5	6.47	2
231.99	6	8.62	2
235.60	7	7.43	2
240.15	8	6.93	2
243.28	9	3.24	1
243.30	10	4.00	2
244.20	11	1.87	1
244.96	12	6.57	2
250.60	13	3.40	1
250.71	14	4.02	2
250.82	15	8.07	2
252.28	16	5.37	2
254.37	17	4.27	2
258.25	18	5.05	2
265.03	19	8.95	2
266.55	20	2.19	1
266.89	21	4.97	2
270.69	22	1.57	1
271.36	23	8.13	2
271.41	24	4.93	2
275.40	25	4.41	2
278.96	26	3.02	1
279.11	27	2.55	1
283.72	28	9.57	2
285.20	29	9.22	2
286.16	30	8.97	2
289.00	31	6.78	2
289.38	32	5.80	2
290.72	33	6.90	2
291.66	34	6.81	2
293.70	35	9.53	2
296.04	36	5.38	2
296.25	37	9.60	2
296.28	38	4.26	2
297.48	39	4.58	2
298.44	40	8.56	2

Note: Group number, 1: Blank control group (C group); 2: Modeling groups (MC, BI, SI, CI Groups).

**Table S2:** Random number table after modeling

**Weight (g)**	**Serial number**	**Random numbers**	**Group number**
220.21	1	8.49	4
221.32	2	4.20	2
224.00	3	5.45	2
227.20	4	5.52	3
231.99	5	8.60	2
235.60	6	3.23	4
240.15	7	7.10	1
243.30	8	2.49	3
244.96	9	3.93	2
250.71	10	6.60	1
250.82	11	3.41	4
252.28	12	9.98	2
254.37	13	8.87	1
258.25	14	7.23	3
265.03	15	2.79	2
266.89	16	6.51	4
271.36	17	2.02	4
271.41	18	6.08	3
275.40	19	7.17	1
283.72	20	6.08	1
285.20	21	5.65	3
286.16	22	8.88	4
289.00	23	4.10	1
289.38	24	1.67	3
290.72	25	8.49	3
291.66	26	4.20	1
293.70	27	5.45	4
296.04	28	7.06	3
296.25	29	5.52	2
296.28	30	8.60	4
297.48	31	3.23	2
298.44	32	7.10	1

Note: Group number, 1: Modeling control group (MC group); 2: BTX-A intervention group (BI group); 3: SPS intervention group (BI group); 4: SPS combined with BTX-A intervention (CI group).

**Table S3:** Four-grade scoring scale for synovial tissue histopathological diagnosis

**Score**	**Degree**	**Definition of scoring**
0 (−)	Normal	Under the experimental conditions, combined with factors such as age, sex, and species, changes may occur, but in other cases, it may be considered normal.
1 (+)	Slight	Changes are minimal and barely exceed the normal range (i.e., minimal change).
2 (++)	Mild	Lesions are easily identifiable but limited in severity. They may not cause any functional impairment, affecting 11%–20% of the examined tissues.
3 (+++)	Moderate	Lesions are prominent and likely to develop in severity. May cause limited tissue or organ dysfunction, involving 21%–40% of the examined tissues.
4 (++++)	Severe	Severe Lesions are severe, with complete lesion formation, leading to expected tissue or organ dysfunction, affecting 41%–100% of the examined tissues.

## Data Availability

The data for this study can be obtained from the corresponding author upon reasonable request.
